# Contact Heat Evoked Potentials Are Responsive to Peripheral Sensitization: Requisite Stimulation Parameters

**DOI:** 10.3389/fnhum.2019.00459

**Published:** 2020-01-10

**Authors:** Lukas D. Linde, Jenny Haefeli, Catherine R. Jutzeler, Jan Rosner, Jessica McDougall, Armin Curt, John L. K. Kramer

**Affiliations:** ^1^ICORD, The University of British Columbia, Vancouver, BC, Canada; ^2^School of Kinesiology, The University of British Columbia, Vancouver, BC, Canada; ^3^Brain and Spinal Injury Center, Department of Neurosurgery, University of California, San Francisco, San Francisco, CA, United States; ^4^Spinal Cord Injury Center, Balgrist University Hospital, University of Zurich, Zurich, Switzerland; ^5^Department of Neurology, University Hospital Bern, Inselspital, University of Bern, Bern, Switzerland; ^6^School of Rehabilitation Sciences, Faculty of Medicine, The University of British Columbia, Vancouver, BC, Canada

**Keywords:** capsaicin, contact heat evoked potentials, type II A mechanoheat nociceptors, EEG, hyperalgesia

## Abstract

The sensitizing effect of capsaicin has been previously characterized using laser and contact heat evoked potentials (LEPs and CHEPs) by stimulating in the primary area of hyperalgesia. Interestingly, only CHEPs reveal changes consistent with notion of peripheral sensitization (i.e., reduced latencies). The aim of this study was to investigate contact heat stimulation parameters necessary to detect peripheral sensitization related to the topical application of capsaicin, and therefore significantly improve the current method of measuring peripheral sensitization via CHEPs. Rapid contact heat stimulation (70°C/s) was applied from three different baseline temperatures (35, 38.5, and 42°C) to a 52°C peak temperature, before and after the topical application of capsaicin on the hand dorsum. Increased pain ratings in the primary area of hyperalgesia were accompanied by reduced N2 latency. Changes in N2 latency were, however, only significant following stimulation from 35 and 38.5°C baseline temperatures. These findings suggest that earlier recruitment of capsaicin-sensitized afferents occurs between 35 and 42°C, as stimulations from 42°C baseline were unchanged by capsaicin. This is in line with reduced thresholds of type II A-delta mechanoheat (AMH) nociceptors following sensitization. Conventional CHEP stimulation, with a baseline temperature below 42°C, is well suited to objectively detect evidence of peripheral sensitization.

## Introduction

Approximately one in five individuals live with chronic pain (Schopflocher et al., 2011; Kuehn, 2018), with a combined economic impact greater than cancer, HIV, and heart disease combined (Phillips, 2009). The sensitization of nociceptive neurons in the periphery is often a key first step in the development of persistent and chronic pain (Latremoliere and Woolf, 2009). The study of chronic pain, and the development of sensitization, has long relied on the experimental induction of pain via controlled noxious stimuli, such as capsaicin (Basbaum et al., 2009; Latremoliere and Woolf, 2009; Woolf, 2011). The topical application of capsaicin results in a predictable area of primary hyperalgesia (LaMotte et al., 1991; Kilo et al., 1994), a key indicator of peripheral sensitization. The sensitizing effect of capsaicin is readily detected in humans as reduced heat pain thresholds and increased sensitivity to mechanical stimuli. Based on its robust, reversible, and minimally-invasive nature, capsaicin represents a widely popular, translational pain model to investigate analgesic drug properties and neuromodulatory effects (Kazarinov et al., 1977; Poyhia and Vainio, 2006; Jutzeler et al., 2015; Larsen et al., 2018; Vollert et al., 2018).

The underlying mechanisms of capsaicin induced primary hyperalgesia are attributable to sensitization of transient receptor potential cation channel subfamily V member 1 (TRPV-1) (Rosenbaum and Simon, 2007). Specific fiber types sensitized by capsaicin include type II AMH nociceptors (Ringkamp et al., 2001; Dubin and Patapoutian, 2010). These afferents are responsible for conveying “first pain,” typically recruited following thermal stimulation at or above 42°C (Treede et al., 1995, 1998; Harkins et al., 2000; Arendt-Nielsen and Chen, 2003), and commonly investigated in humans using contact heat and laser evoked potentials (CHEPs and LEPs) (Harkins et al., 2000; Chen et al., 2001; Iannetti et al., 2006).

In line with behavioral signs of sensitization, CHEPs are modulated following application of capsaicin and stimulation in the primary area of hyperalgesia. This is evidenced as increased pain ratings and reductions in N2 waveform latency (Madsen et al., 2012; Jutzeler et al., 2015). However, an understanding of latency reductions and neural processes involved remains unknown.

One possibility for N2 latency reductions in response to capsaicin is that type II AMH afferents are temporally recruited earlier in the periphery during contact heat stimulation. Conventionally, contact heat stimulation is delivered from a low (e.g., 35°C) baseline to a peak temperature (e.g., 52°C) at a fixed, nominal rate (e.g., 70°C/s) (Kramer et al., 2012a, b, 2013, 2016; Haefeli et al., 2013, 2014b). This means that stimulation passes through lower temperatures before activating type II AMH nociceptors (at ≥∼42°C) (Baumgartner et al., 2005). After the topical application of capsaicin, type II AMH nociceptor threshold is reduced (e.g., to 38–40°C), which results in earlier onset CHEPs (i.e., reduced latency).

To test this theory, we examined CHEPs in the primary area of hyperalgesia using three different baseline temperatures (i.e., 35, 38.5, and 42°C). Elevated baseline temperatures were intended to decrease recruitment of afferents by contact heat stimulation below the normal threshold of type II AMH nociceptors (∼42–46°C) (Treede et al., 1995, 1998; Harkins et al., 2000; Arendt-Nielsen and Chen, 2003). We hypothesized that applying baseline temperatures of 38.5 and 42°C would attenuate reductions in N2 latency compared to 35°C baseline stimulation. Our findings will improve the understanding of neuromodulatory influences of CHEPs N2 latencies in the assessment of peripheral sensitization in humans.

## Materials and Methods

### Subjects

Thirteen healthy subjects without a history of chronic pain and neurological disease participated in the study. Exclusion criteria comprised pregnancy, intake of any medication (except birth control), and any obvious neurological condition. The experimental protocol conformed to the standards set by the Declaration of Helsinki and was approved by the local ethical committee. All subjects gave written informed consent.

### Experimental Design

**FIGURE 1:**
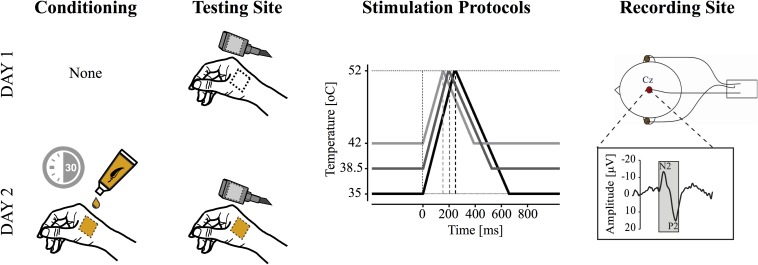
Study design. Contact heat evoked potentials (CHEPs) were acquired on separate testing days (i.e., with and without capsaicin). Capsaicin was applied topically for 30 min prior to CHEPs acquisition on day two. CHEP stimulation was applied from three baseline temperatures: (1) 35°C, (2) 38.5°C, and (3) 42°C to a peak temperature of 52°C. These resulted in stimulus durations of 243, 193, and 143 ms (i.e., onset to peak), respectively. The order of baseline temperature was randomized, but maintained between experimental sessions. All stimuli were delivered to a 52°C peak temperature. A vertex recording electrode (Cz, referenced to linked ears) was used to acquire the most prominent CHEP (i.e., N2P2).

In the current study, all subjects participated in two CHEP recording sessions. Experimental sessions were separated by at least 48 hours, to a maximum of 7 days. In the second recording session, subjects underwent 30 min of topical capsaicin sensitization prior to contact heat stimulation (Figure 1).

### Contact Heat Stimulation

A contact heat stimulator (PATHWAY Pain and Sensory Evaluation System; Medoc, Ramat Yishai, Israel) was used to deliver noxious heat stimuli. The thermode (27 mm diameter with a stimulating surface of 572.3 mm^2^) is composed of a heating thermofoil covered with a thermo conductive plastic. The thermofoil allows a heating rate of 70°C/s and is actively cooled by a peltier element (40°C/s). Contact heat stimuli were applied in an area [approximately 16 cm^2^ (1600 mm^2^)] within the boundaries of the C6 dermatome, on the dorsum surface, at the base of the thumb from three baseline temperatures (35, 38.5, and 42°C) in random order. These were selected to represent starting points below (35 and 38.5°C) and at/above type II AMH nociceptor thresholds (∼42°C). The order of stimulation was randomized between subjects and maintained across the first and second experimental session. Irrespective of the baseline temperature, the target peak stimulation intensity was set to 52°C. The different baseline temperature conditions are displayed in Figure 1. For each condition, 10 stimulations (Kramer et al., 2013, 2016) were applied with an 8–12 s inter-pulse interval (Jutzeler et al., 2016; Rosner et al., 2018). A low number of stimulations was used to avoid peripheral sensitization caused by repetitive cutaneous stimulation. A total of 30 stimulations were performed on each testing day. After each contact heat stimuli, the thermode was repositioned within a 16 cm^2^ (1600 mm^2^) boundary (i.e., lifted from the skin and placed at a different location within the outlined area) to reduce receptor fatigue (Greffrath et al., 2007). As a result, no two consecutive stimulations were performed in exactly the same area. The same procedure was performed in the capsaicin as in the no-capsaicin condition. Subjects rated the perceived intensity using a numeric rating scale ranging from 0 to 10 after each stimulus (0-no pain, 10-worst pain imaginable). Subjects were asked to keep their eyes open during contact heat stimuli and blink, if necessary, to an acoustic cue presented 4 s after stimulation.

### Capsaicin Application

Capsaicin was applied preceding contact heat stimulation in the second CHEP recording session. One ml of 0.075% capsaicin cream (Hänseler, Herisau, Switzerland) was applied to a 16 cm^2^ area within the boundaries of the C6 dermatome. Similar concentrations of capsaicin have been applied previously to induce experimental pain (Srbely et al., 2010; Andresen et al., 2011; Jutzeler et al., 2015; Linde and Srbely, 2019). The 16 cm^2^ (1600 mm^2^) area was limited by tape. During capsaicin sensitization, subjects were asked to rate their pain on a numeric rating scale from 0 to 10 every 5 min. After 30 min of capsaicin sensitization, the remaining cream was removed.

### CHEP Recording

For the electroencephalography scalp recording sites were prepared with alcohol and Nuprep (D.O. Weaver and Company, Aurora, CO, United States). Gold cup electrodes were positioned on Cz and referenced to linked earlobes (Cz-A1-A2) to record N2 and P2 waveforms. A wet ground strap was attached to the subjects’ forearm. We used a reduced electrode set up as numerous previous studies have reliably produced N2 and P2 waveforms from the Cz electrode referenced to linked earlobes (Treede et al., 1988; Bromm and Chen, 1995; Kakigi et al., 2004; Wydenkeller et al., 2008; Kramer et al., 2012a, b; Albu et al., 2013; Jutzeler et al., 2015, 2016; Rosner et al., 2018). CHEPs were sampled at 2000 Hz using a preamplifier (20000×, bandpass filter 0.25–300 Hz; ALEA Solutions, Zurich, Switzerland). Data were recorded in a Labview based program (V1.43 CHEP; ALEA Solutions, Zurich, Switzerland) using a time-frame of 100 ms pre-trigger and 1000 ms post-trigger. Data were bandpass filtered offline with a 0.5–30 Hz filter.

### Data Analysis

EEG Epochs from −100 pre-trigger to +1000 ms post-trigger were visually analyzed by a blinded and experienced examiner (JH), without knowledge of stimulation conditions or the baseline temperatures. A minimum of nine artifact (e.g., blink) free trials was included for N2 and P2 waveform averaging within each condition for each participant. The average number of trials included in CHEPs waveform averaging was 9.81 ± 0.40 (Mean ± SD). Two separate examiners (CJ and JK) confirmed N2 and P2 waveforms. Disagreement was discussed between examiners until consensus was reached. CHEPs parameters (N2 latency, N2 amplitude, P2 latency, P2 amplitude, and N2P2 amplitude) were calculated from averaged waveform data.

All statistical analyses were performed in R (Version 3.5.3, MacOs Mojave). The skewed distribution of CHEP parameters was corrected by log transformation. A linear mixed effects model with repeated measures was first applied to examine the relationship between time and perceived intensity of capsaicin application over 30 min. A second linear mixed effects model examined the main effects of baseline temperature (35, 38.5, and 42°C) and capsaicin application on log-transformed CHEP outcomes. Interactions between baseline temperature and capsaicin were examined to delineate whether baseline temperature had a differential effect on CHEPs outcomes. The model was adjusted for the stimulation order. *Post hoc* pairwise comparisons were Bonferroni corrected. Statistical significance was set at α = 0.05. All of the data used in our analysis, extracted from CHEP waveforms, is shown in Supplementary Table S1.

## Results

One subject could not tolerate any contact heat stimulation during application with capsaicin and was excluded from analysis. The average age of the remaining 12 subjects (10 males) was 31.5 ± 7.7 years (± SD).

### Capsaicin Sensitization Period

**FIGURE 2:**
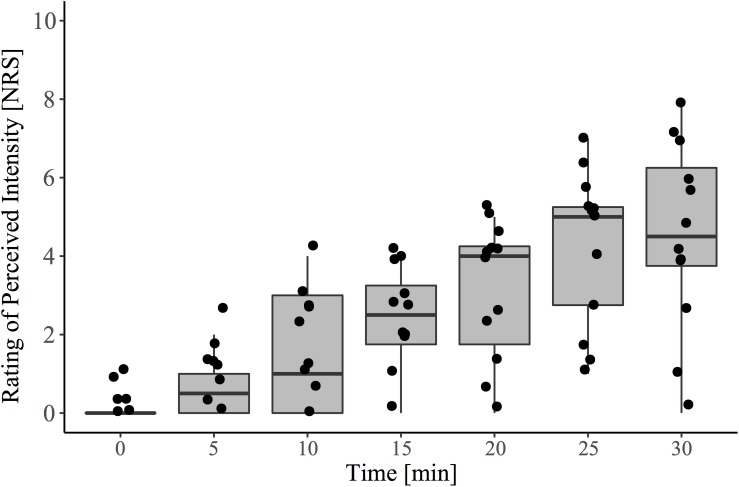
Pain ratings to capsaicin exposure over the 30-minute conditioning period (i.e., pre-contact heat stimulation). During this time, participants sat quietly and were instructed to rate spontaneously perceived intensity (0–10) to the area of capsaicin application every 5 min. The baseline (time 0) recording represents the reported sensation immediately after capsaicin was applied to the entire area. Contact heat stimulation and the acquisition of evoked potentials were performed at 30 min, following cream removal.

In agreement with the known effects of topically applied 0.075% capsaicin cream, there was a significant main effect of time on pain intensity rating (β = 0.155, CI [95%]: 0.13 – 0.18, *p* < 0.001) (Figure 2).

### Main Effect of Capsaicin Sensitization

**FIGURE 3:**
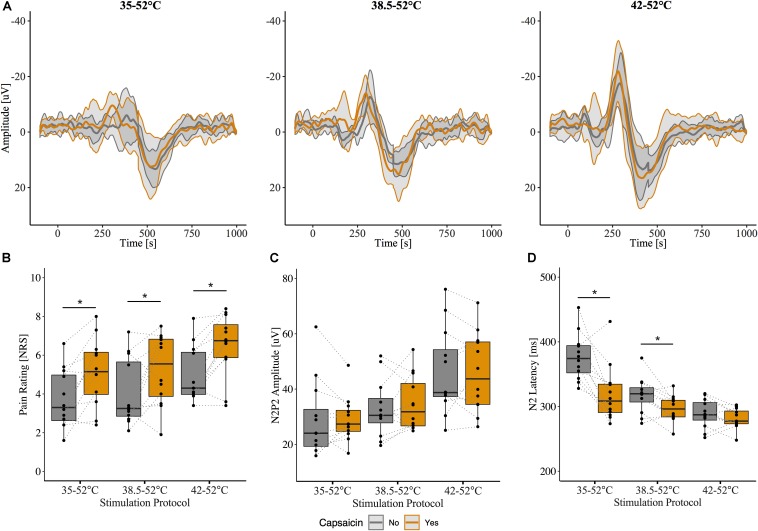
Effect of capsaicin on contact heat evoked potentials (CHEPs). **(A)** Grand-average N2P2 waveforms from all subjects (*n* = 12). Shaded area represents between subject standard deviation. **(B)** Pain ratings, **(C)** N2P2 amplitude, **(D)** N2 latency before and after capsaicin. Pain ratings were significantly increased following capsaicin application for all baseline temperatures. N2 latency was significantly affected by capsaicin and only when stimulation was performed from 35 and 38.5°C baseline temperatures. ^∗^ Denotes a significant difference between capsaicin “Yes” and “No” sessions for respective baseline temperatures, *p* < 0.05.

**FIGURE 4:**
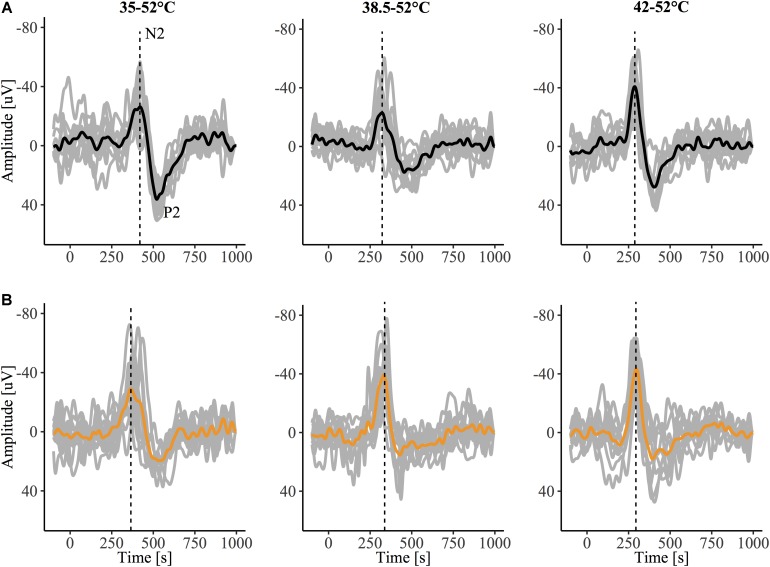
Contact heat evoked potentials (CHEPs) from a representative subject before **(A)** and after **(B)** capsaicin sensitization, recorded in response to stimulation from a 35, 38.5, and 42°C baseline. The dotted vertical line denotes N2 latency, observed to be earlier in the 35°C baseline condition following capsaicin sensitization in this representative tracing.

The grand-average CHEPs for all baseline temperature condition with and without capsaicin sensitization are illustrated in Figure 3A. A representative example is illustrated in Figure 4.

Overall, pain ratings to the contact heat stimulation were significantly higher after the capsaicin sensitization (β = 0.34, CI [95%]: 0.17 – 0.51, *p* < 0.001) (Figure 3B). This is consistent with the known effect of capsaicin to induce hyperalgesia to heat in the primary area of application. CHEP amplitudes (i.e., N2P2, N2, and P2) and P2 latency were unaffected by capsaicin [N2P2 amplitude: β = 0.07, CI [95%]: −0.08 – 0.22, *p* = 0.365 (Figure 3C); N2 amplitude: β = 0.10, CI [95%]: −0.09 – 0.29, *p* = 0.296; P2 amplitude: β = 0.02, CI [95%]: −0.16 – 0.21, *p* = 0.823; P2 latency β = −0.05, CI [95%]: −0.10 – 0.00, *p* = 0.082]. Overall, N2 latency was significantly reduced after capsaicin sensitization (β = −0.17, CI [95%]: −0.22 – −0.12, *p* < 0.001) (Figure 3D).

### Interaction of Baseline Temperature Condition and Capsaicin Application

There was a significant interaction effect between baseline temperature condition and capsaicin sensitization for N2 latency (42°C: β = 0.14, CI [95%]: 0.07 – 0.21, *p* < 0.001). This suggests that the reduction in N2 latency depended on the baseline temperature. At 35°C and 38.5°C baseline temperatures, N2 latency was significantly shorter after capsaicin sensitization (35°C: β = −0.17, CI [95%]: −0.24 – −0.10, *p* < 0.001; 38.5°C: β = −0.07, CI [95%]: −0.11 – −0.02, *p* = 0.011). Only one subject did not demonstrate a decreased N2 latency following the application of capsaicin and stimulation from a 35°C baseline temperature. From a 38.5°C baseline temperature, only three subjects demonstrated a nominal increase in N2 latency. N2 latencies were comparable before and after capsaicin for 42°C baseline condition (β = −0.03, CI [95%]: −0.06 – 0.01, *p* = 0.166; Figure 3D). There were no interaction effects (baseline temperature condition and capsaicin) for N2P2 amplitude or P2 latency. Model summaries from the statistical analysis are shown in Supplementary Tables S2, S3.

### Addendum to Results

We acquired CHEPs from an additional six participants (3 males, age 25 ± 3.5 years) to confirm that the number of stimulations used in CHEPs acquisition was not a limitation to our findings. Six participants completed study procedures as outlined above; the only methodological difference was that participants received 20 contact heat stimuli per baseline temperature as opposed to 10 stimuli. EEG data was recorded from 32 active electrodes via international 10–20 positioning (REF) (Brain Vision LLC, Morrisville, NC, United States). CHEPs waveforms were calculated from the Cz vertex position, referenced to linked earlobes. N2 latencies were calculated from averaged waveform data using the first 10 stimuli and all 20 stimuli of EEG epochs 100 ms pre-stimulus to 1000 ms post-stimulus. All CHEPs waveforms were visually inspected for artifacts (i.e., blink). The average number of trials used in CHEPs waveform averaging was 19.77 ± 0.76 for 20 stimuli and 9.88 ± 0.34 for 10 stimuli, respectively. N2 latencies were compared between 10 and 20 stimuli methods of calculation, using two-sample *t*-tests. We observed no significant differences between N2 latencies calculated from 10 stimuli vs. 20 stimuli for all baseline temperature stimulation protocols (35°C: 20 stimuli – 361 ms, CI [95%]: 338–381; 10 stimuli – 366 ms, CI [95%]: 346–385, *p* = 0.72; 38.5°C: 20 stimuli – 302 ms, CI [95%]: 289–316; 10 stimuli – 291 ms, CI [95%]: 283–298, *p* = 0.20; 42°C: 20 stimuli – 268 ms, CI [95%]: 261–276; 10 stimuli – 265 ms, CI [95%]: 255–276, *p* = 0.71) (Supplementary Table S4).

**FIGURE 5:**
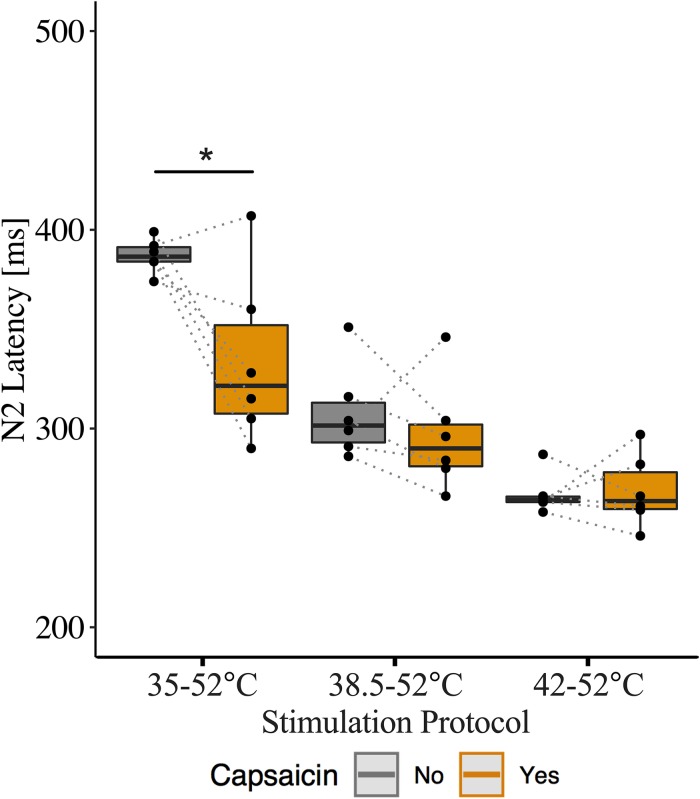
Effect of capsaicin on contact heat evoked potentials (CHEP) N2 latency with stimulations delivered at 35, 38.5, and 42°C baselines. Data displayed from additional six participants, that underwent 20 stimulations per condition as opposed to 10 stimulations. ^∗^ Denotes a significant difference between capsaicin “Yes” and “No” sessions for respective baseline temperatures, *p* < 0.05.

We also examined the influence of capsaicin on N2 latency between baseline temperatures for this additional *N* = 6 dataset. Similar to our original findings, we observed a main effect of capsaicin sensitization on N2 latency (β = −0.15, CI [95%]: −0.23 – −0.07, *p* = 0.001) as well as an interaction effect of capsaicin and baseline temperature (42°C: β = 0.16, CI [95%]: 0.05 – 0.27, *p* = 0.01). This interaction effect suggests that the reduction in N2 latency depended on the baseline temperature. At the 35°C baseline temperature, N2 latency was significantly reduced after capsaicin sensitization (β = −0.15, CI [95%]: −0.24 – −0.05, *p* = 0.014). At 38.5 and 42°C baseline temperatures, there was no significant effect of capsaicin on N2 latency (38.5°C: β = −0.04, CI [95%]: −0.12 – 0.04, *p* = 0.364; 42°C: β = 0.00, CI [95%]: −0.05 – 0.06, *p* = 0.892) (Supplementary Table S5). Only 1 of 6 participants did not demonstrate a decreased N2 latency following the application of capsaicin and stimulation for both 35 and 38.5°C baseline temperatures, however, the effect of capsaicin did not reach statistical significance for the 38.5°C baseline temperature. Similar to our original findings, N2 latencies were comparable before and after capsaicin application for the 42°C baseline temperature (Figure 5).

## Discussion

As in previous CHEP studies applying conventional baseline temperature stimulation (Madsen et al., 2012; Jutzeler et al., 2015), N2 latency was reduced and pain rating increased following stimulation in the primary area of hyperalgesia. The effect of sensitization on N2 latency was significant when stimulation at 35 and 38.5°C baselines. Increasing the baseline temperature to 42°C attenuated latency reductions, to the point that N2 latency was not significantly different following sensitization. These findings were replicated in our additional dataset, with significant reductions in N2 latency observed at 35°C baseline, non-significant reductions in N2 latency at 38.5°C in five of six additional participants, and comparable N2 latencies following capsaicin application for 42°C baseline. Together, these findings from both our original and additional dataset suggest that recruitment of capsaicin-sensitized afferents occurs below 42°C.

N2 latencies obtained during the control session from 35°C and 42°C baseline temperature conditions were in line with previously reported normative values for the C6 dermatome (Jutzeler et al., 2016). The N2 latencies following capsaicin application in the 35°C baseline condition [319.8 ± 43.9 ms (mean ± SD)] were significantly earlier than the control condition (376.8 ± 45.1 ms) and were also earlier than previously reported normal values for N2 latencies (384.1 ± 31.9 ms) (Jutzeler et al., 2016). This trend was replicated in our additional validation dataset, with pre-capsaicin N2 latencies (387.0 ± 8.5 ms) in line with previously reported normal values (384.1 ± 31.9 ms) and post capsaicin N2 latencies (334.2 ± 42.8 ms) shifting to precede previously reported normal values (Jutzeler et al., 2016). Together, these findings suggest the effect of capsaicin on N2 latency, acquired via 35°C baseline, was both statistically significant and clinically relevant, as sensitized N2 latencies occurred earlier than previously reported normal values.

Our findings also clearly demonstrate that reduced N2 latency is not related to thermal hyperalgesia *per se*. This was evidenced by the fact that pain ratings significantly increased in the primary area of hyperalgesia for all baseline temperature stimulations, even in the absence of reduced N2 latency (i.e., 42°C baseline temperature CHEP stimulation). This temperature dependent dichotomy of the effect of capsaicin on pain ratings and N2 latencies in response to contact heat may suggest separate mechanisms of peripheral sensitization. Previous studies in both humans and animals have suggested primary hyperalgesia to be mediated by the sensitization of C-fiber nociceptors, while secondary hyperalgesia is mediated by sensitized A-fiber nociceptors (Culp et al., 1989; Hsieh et al., 2015). Given our stimulation site was within the area of primary hyperalgesia and given the neural pathway of CHEPs activation via type II AMH nociceptors (Chen et al., 2001; Iannetti et al., 2006; Truini et al., 2007), a possible explaination for our findings may be that pain ratings were influenced by C-fiber sensitzation, while reductions in N2 latencies were mediated by the sensitiztion of type II AMH nociceptors. Future studies should explore this avenue further through the use of nerve block or compression ischemia testing during CHEPs acquisition with capsaicin.

There is little debate that the neural signature of contact heat stimulation (i.e., vertex N2P2 waveform) arise from activation of type II AMH nociceptors (Chen et al., 2001; Iannetti et al., 2006; Truini et al., 2007). It logically follows that sensitizing type II AMH nociceptors with capsaicin should lead to profound changes in CHEPs. Our findings replicate those of previous CHEP studies using similar stimulation parameters (35 to 52°C at 70°C/s) (Madsen et al., 2012; Jutzeler et al., 2015), in that N2 latency was reduced and pain rating increased in the primary area of hyperalgesia. The most pragmatic explanation for a reduction in N2 latency is that capsaicin modulates type II AMH nociceptors, lowering their activation threshold to thermal stimulation. From lower baseline temperatures, threshold is reached faster, temporally recruiting the same or similar population of thinly myelinated afferents earlier in the heat stimulus.

Consistent with the notion of earlier recruitment, stimulation from more conventional baseline temperatures (e.g., 35°C) was a requisite parameter to detect significant reductions in N2 latency. Higher temperatures failed to reveal the sensitizing effect of capsaicin. From a 42°C baseline, recruitment of type II AMH nociceptors may already occur as fast as is physiologically possible. Recruitment occurs at or above the normal threshold for activation of type II AMH nociceptors, creating a “floor effect” that prevents the measurement of peripheral sensitization. Overall, these observations suggest that stimulation between 35 and 42°C is key to detecting peripherally sensitized type II AMH afferents, which coincides with behavioral evidence of sensitization below 40°C (Schaldemose et al., 2015).

Contact heat stimulation is now widely regarded as sufficient for the acquisition of nociceptive evoked potentials. CHEP stimulation slowly activates nociceptors, sequentially, from a starting baseline temperature below the recruitment threshold of type II AMH afferents (e.g., 35°C). As is evident in Figure 4, even a low number of stimulations (10) yield a larger and reliable vertex waveform, particularly at higher baseline temperatures. This is in line with previous CHEPs studies (Kramer et al., 2013, 2016), and was further demonstrated in our own comparison between N2 latencies calculated from 10 stimuli to 20 stimuli, respectively. However, compared to other forms of noxious stimulation (e.g., laser), evoked potentials arising from contact heat stimulation are less synchronized and subject to greater temporal dispersion and higher latency jitter (variability in latency among averaged trials) (Granovsky et al., 2008). Nevertheless, contact heat stimulation may present an opportunity to specifically and objectively assess the role of peripheral sensitization (i.e., lowering of type II AMH nociceptor thresholds below 42°C). Future studies directly comparing laser and CHEPs are needed to comprehensively evaluate sensitivity and optimal stimulation parameters to detect primary hyperalgesia.

Our control observations (i.e., changing baseline temperature in the absence of capsaicin) demonstrate an obvious advantage of increasing the baseline temperature to acquire CHEPs N2P2 amplitudes. This confirms our previous findings (Kramer et al., 2012a, 2013; Haefeli et al., 2014a). The impact of increasing the baseline temperature on contact heat evoked potentials is chiefly a function of increasing correspondence between the theoretical and acute peak temperature experienced at the nociceptor. From a 35°C baseline, at 70°C/s, only a fraction of the nominal peak temperature is reached at the level of the nociceptors (Baumgartner et al., 2005). This represents a well-known technical challenge, which is due to the temperature of the skin lagging behind the temperature of the thermode. As a result, the thermode returns to baseline (i.e., actively cooling) before the skin (or the nociceptor) ever reaches the desired peak temperature. Shifting the starting point of contact heat stimulation to higher temperatures simply means that higher peak temperatures can be physiologically achieved. This, in turn, recruits a larger number of afferents, yielding higher pain ratings and increased CHEP amplitudes. Other factors, like improving synchronization of the afferent volley by shortening the stimulus duration, may also play a role in generating larger amplitude evoked potentials (Iannetti et al., 2004).

An alternative explanation for our results is that peripheral sensitization shifted recruitment to larger and faster conducting type II AMH afferents. From a 35°C baseline temperature, a reduction in CHEP latency occurs because control stimulation recruits slower conducting afferents. Higher baseline temperatures fail to convey a change in latency shift, chiefly because the fastest conducting afferents are already maximally recruited in the absence of peripheral sensitization (i.e., “floor effect”). Against such a proposal, our observed change in N2 latency from 35 to 38.5 and 42°C is almost exactly as predicted by changes in stimulation duration accompanying the increase in baseline temperature (i.e., nominal change in N2 latency predicted by a change in stimulus duration 35 to 38.5°C = −50 ms, actual change = −59 ± 25 ms; nominal change in stimulus duration from 35 to 42°C = −100 ms, actual change = −88 ± 30 ms; values are shown as averages ± standard deviations). Additionally, a recent study reported similar reductions in N2 latency (341 ± 90 ms – 285 ± 34 ms) with CHEPs baseline temperature increases from 35 to 40°C (Nakata et al., 2018), albeit non-significant. Overall, this suggests that similar populations of type II AMH nociceptors are recruited, across baseline temperatures (i.e., under control conditions).

These observations are limited to the temporal representation of the vertex N2P2 waveform. Time-frequency analyses may reveal non-phase locked responses that correspond with behavioral evidence of primary hyperalgesia. While stimulation conditions were randomized, capsaicin exposure always followed a recording session without capsaicin. This was done to avoid carry-over effects of capsaicin but may have influenced the findings. However, randomization of recording sessions would not overcome the inability to successfully blind participants to the perceptual effects of capsaicin, a known limitation of the capsaicin model. Previous studies have demonstrated fair to excellent test-retest reliability of CHEPs N2 latencies from cervical dermatomes (Kramer et al., 2012b) and lumbar dermatomes (Rosner et al., 2018), providing further confidence that our observed reductions in N2 latencies were due to the effects of capsaicin. Finally, a relatively low number of subjects (*n* = 12) may have contributed to type II error in relation to some of our observations (e.g., changes in amplitude). While our findings are limited to a small sample size (*n* = 12), we demonstrated the ability to successfully replicate our findings with a separate cohort of six participants. Nevertheless, the effect of capsaicin on N2 latency was robust.

## Conclusion

In conclusion, capsaicin sensitization resulted in significant reductions in N2 latency when contact heat was delivered from a conventional baseline temperature (35 and 38.5°C). This effect is attributed to a reduction in activation threshold of type II AMH nociceptors and stimulation between 35 and 42°C, as stimulations from 42°C baseline were unchanged by capsaicin. CHEPs may be useful as a measure of peripheral sensitization related to the topical application of capsaicin, aiding in the evaluation of pain mechanisms.

## Data Availability Statement

The original data (raw EEG traces) are available from the corresponding author upon reasonable request.

## Ethics Statement

The studies involving human participants were reviewed and approved by local ethics boards of the University of British Columbia and the University of Zurich. The patients/participants provided their written informed consent to participate in this study.

## Author Contributions

All authors assisted in the interpretation of the data, and the writing and editing of the manuscript. JH collected the data. CJ performed the original analysis and created the figures. LL collected the additional dataset and performed subsequent re-analysis.

## Conflict of Interest

The authors declare that the research was conducted in the absence of any commercial or financial relationships that could be construed as a potential conflict of interest.

## Abbreviations

AMH: A-delta mechanoheat nociceptors
CHEPs: contact heat evoked potentials
LEPs: laser evoked heat potentials
TRPV-1: transient receptor potential cation channel subfamily V member 1.
