# Supplementary material for: Contact Heat Evoked Potentials Are Responsive to Peripheral Sensitization: Requisite Stimulation Parameters
Source: Front Hum Neurosci. 2020 Jan 10;13:459. doi: 10.3389/fnhum.2019.00459 (PMC6966714; doi:10.3389/fnhum.2019.00459)
Supplement: Supplementary file 1 [file Table_1.DOCX]

**Supplementary Table 1:** Subject characteristics and individual CHEPs parameters for each baseline stimulation protocol with and without capsaicin sensitization.

| ID | Stimulation Order | Capsaicin [yes/no] | Baseline Temperature [ºC] | Pain Rating [NRS] | N2P2 Amplitude [uV] | N2 Amplitude [uV] | P2 Amplitude [uV] | N2 Latency [ms] | P2 Latency [ms] |
| --- | --- | --- | --- | --- | --- | --- | --- | --- | --- |
| C01 | 3 | No | 35 | 2.4 | 15.9 | -8.8 | 7.1 | 361.5 | 482 |
| C02 | 1 | No | 35 | 2.9 | 62.5 | -26.2 | 36.3 | 420.5 | 521 |
| C03 | 1 | No | 35 | 3.2 | 15.9 | -8.3 | 7.6 | 453 | 589 |
| C04 | 2 | No | 35 | 2.7 | 19.8 | -7.4 | 12.3 | 377.5 | 574 |
| C05 | 3 | No | 35 | 5.4 | 39.5 | -13.1 | 26.4 | 355 | 525 |
| C06 | 1 | No | 35 | 6.6 | 30.4 | -15.1 | 15.2 | 328 | 510 |
| C07 | 2 | No | 35 | 5.2 | 24.1 | -11 | 13.1 | 393.5 | 548.5 |
| C08 | 2 | No | 35 | 3.4 | 21.4 | -9.9 | 11.5 | 384.5 | 566.5 |
| C09 | 2 | No | 35 | 2.4 | 45 | -20.5 | 24.5 | 396 | 511.5 |
| C10 | 2 | No | 35 | 4 | 24 | -12 | 12 | 343.5 | 397.5 |
| C11 | 3 | No | 35 | 1.6 | 17.8 | -6.5 | 11.2 | 371 | 548.5 |
| C12 | 1 | No | 35 | 4.9 | 28.9 | -11.7 | 17.2 | 337.5 | 543.5 |
| C01 | 2 | No | 38.5 | 2.7 | 29.4 | -16.5 | 12.9 | 290.5 | 395.5 |
| C02 | 2 | No | 38.5 | 2.9 | 40.6 | -22.9 | 17.6 | 321.5 | 455 |
| C03 | 3 | No | 38.5 | 2.6 | 20.9 | -14.4 | 6.5 | 375 | 489 |
| C04 | 1 | No | 38.5 | 3.1 | 35.3 | -18.5 | 16.8 | 319 | 508 |
| C05 | 1 | No | 38.5 | 5.5 | 49.9 | -17.7 | 32.2 | 338 | 452 |
| C06 | 3 | No | 38.5 | 7.2 | 19.6 | -12 | 7.6 | 274 | 446 |
| C07 | 3 | No | 38.5 | 6.1 | 30.4 | -17.7 | 12.7 | 337 | 486.5 |
| C08 | 3 | No | 38.5 | 3.2 | 23 | -11.4 | 11.6 | 312.5 | 522.5 |
| C09 | 1 | No | 38.5 | 3.3 | 52 | -30.8 | 21.2 | 312.5 | 448.5 |
| C10 | 1 | No | 38.5 | 3.4 | 30.2 | -15.9 | 14.3 | 281 | 397.5 |
| C11 | 2 | No | 38.5 | 2.1 | 32.4 | -14.7 | 17.8 | 326.5 | 462.5 |
| C12 | 2 | No | 38.5 | 6.2 | 30.6 | -15.7 | 14.9 | 320.5 | 429 |
| C01 | 1 | No | 42 | 4.6 | 49.9 | -29.4 | 20.6 | 270 | 400.5 |
| C02 | 3 | No | 42 | 4 | 68.5 | -40.8 | 27.7 | 286.5 | 405 |
| C03 | 2 | No | 42 | 4.1 | 25.1 | -16.9 | 8.2 | 311 | 504 |
| C04 | 3 | No | 42 | 3.4 | 37.8 | -16.1 | 21.7 | 288 | 455.5 |
| C05 | 2 | No | 42 | 6.1 | 60.4 | -22.6 | 37.8 | 282 | 396 |
| C06 | 2 | No | 42 | 7.9 | 38.6 | -17.5 | 21.1 | 252 | 450.5 |
| C07 | 1 | No | 42 | 6.3 | 38.6 | -28.2 | 10.4 | 304.5 | 468 |
| C08 | 1 | No | 42 | 3.9 | 30.4 | -18.5 | 11.9 | 320 | 474.5 |
| C09 | 3 | No | 42 | 4.3 | 52.2 | -23.6 | 28.5 | 284 | 425.5 |
| C10 | 3 | No | 42 | 3.9 | 38.9 | -24.6 | 14.4 | 257 | 339.5 |
| C11 | 1 | No | 42 | 4.3 | 76.1 | -38.9 | 37.2 | 318.5 | 429 |
| C12 | 3 | No | 42 | 6.8 | 35.8 | -19.8 | 16 | 293 | 393 |
| C01 | 3 | Yes | 35 | 5 | 27.4 | -13.4 | 14 | 273.5 | 501.5 |
| C02 | 1 | Yes | 35 | 6.3 | 48.6 | -29 | 19.6 | 365 | 536 |
| C03 | 1 | Yes | 35 | 6 | 27.3 | -17.7 | 9.6 | 312.5 | 446 |
| C04 | 2 | Yes | 35 | 2.4 | 22 | -12 | 10 | 296 | 484 |
| C05 | 3 | Yes | 35 | 6.1 | 35.4 | -12.7 | 22.7 | 336 | 528.5 |
| C06 | 1 | Yes | 35 | 8 | 31.8 | -18.4 | 13.4 | 304.5 | 516 |
| C07 | 2 | Yes | 35 | 5 | 16.8 | -6.2 | 10.6 | 431.5 | 549.5 |
| C08 | 2 | Yes | 35 | 3.6 | 25 | -9.8 | 15.2 | 287 | 501.5 |
| C09 | 2 | Yes | 35 | 2.6 | 33.9 | -11.4 | 22.5 | 322.5 | 537.5 |
| C10 | 2 | Yes | 35 | 5.3 | 26.3 | -12.5 | 13.7 | 292 | 393.5 |
| C11 | 3 | Yes | 35 | 4.1 | 23.9 | -9.1 | 14.8 | 334 | 541.5 |
| C12 | 1 | Yes | 35 | 7.3 | 30.6 | -12.5 | 18.1 | 283.5 | 498 |
| C01 | 2 | Yes | 38.5 | 6.4 | 46.6 | -28.3 | 18.3 | 257.5 | 464 |
| C02 | 2 | Yes | 38.5 | 6.9 | 54.3 | -39 | 15.3 | 332 | 429 |
| C03 | 3 | Yes | 38.5 | 4.5 | 26 | -17 | 9 | 309 | 496 |
| C04 | 1 | Yes | 38.5 | 1.9 | 24.9 | -10.1 | 14.8 | 281.5 | 477 |
| C05 | 1 | Yes | 38.5 | 6.6 | 40.8 | -14.1 | 26.7 | 315 | 449 |
| C06 | 3 | Yes | 38.5 | 7 | 27.6 | -13.9 | 13.7 | 289 | 491.5 |
| C07 | 3 | Yes | 38.5 | 4.7 | 26.1 | -12.5 | 13.6 | 308.5 | 467.5 |
| C08 | 3 | Yes | 38.5 | 3.5 | 26.9 | -11.2 | 15.6 | 284.5 | 482 |
| C09 | 1 | Yes | 38.5 | 3.4 | 45.8 | -25.3 | 20.4 | 311.5 | 521.5 |
| C10 | 1 | Yes | 38.5 | 6.8 | 34.7 | -17 | 17.7 | 286 | 401.5 |
| C11 | 2 | Yes | 38.5 | 4 | 29.3 | -15 | 14.3 | 303.5 | 433.5 |
| C12 | 2 | Yes | 38.5 | 7.5 | 34.3 | -16.8 | 17.5 | 282.5 | 476 |
| C01 | 1 | Yes | 42 | 7.4 | 56.9 | -32.2 | 24.7 | 248 | 423.5 |
| C02 | 3 | Yes | 42 | 6.8 | 61.4 | -43.3 | 18.1 | 297.5 | 394 |
| C03 | 2 | Yes | 42 | 6.7 | 26.4 | -16.7 | 9.7 | 291.5 | 483.5 |
| C04 | 3 | Yes | 42 | 3.4 | 29.4 | -14.6 | 14.8 | 274 | 454.5 |
| C05 | 2 | Yes | 42 | 8.2 | 53.6 | -23.5 | 30.1 | 276 | 394.5 |
| C06 | 2 | Yes | 42 | 8.1 | 34.5 | -20.3 | 14.2 | 270.5 | 443.5 |
| C07 | 1 | Yes | 42 | 7.2 | 34.4 | -20.1 | 14.2 | 302.5 | 477.5 |
| C08 | 1 | Yes | 42 | 5.9 | 47.5 | -29.5 | 18 | 300 | 490.5 |
| C09 | 3 | Yes | 42 | 3.6 | 57.5 | -29 | 28.5 | 277.5 | 414 |
| C10 | 3 | Yes | 42 | 6.6 | 39.9 | -18.5 | 19.4 | 277.5 | 389 |
| C11 | 1 | Yes | 42 | 5.8 | 71.2 | -32.5 | 38.7 | 287 | 406.5 |
| C12 | 3 | Yes | 42 | 8.4 | 37.5 | -17.4 | 20.1 | 271.5 | 381.5 |
