# Supplementary material for: Contact Heat Evoked Potentials Are Responsive to Peripheral Sensitization: Requisite Stimulation Parameters
Source: Front Hum Neurosci. 2020 Jan 10;13:459. doi: 10.3389/fnhum.2019.00459 (PMC6966714; doi:10.3389/fnhum.2019.00459)
Supplement: Supplementary file 2 [file Table_2.DOCX]

| **Supplementary Table 2:** Effect of stimulation protocol, capsaicin conditioning, and stimulation order on CHEPs latency parameters and rating | | | | | | | | | |
| --- | --- | --- | --- | --- | --- | --- | --- | --- | --- |
|  | **N2 Latency [ms]** | | | **P2 Latency [ms]** | | | **Rating of CHEPs** | | |
|  | *B* | *CI* | *p-value* | *B* | *CI* | *p-value* | *B* | *CI* | *p-value* |
| *(Intercept)* | 5.94 | 5.88 – 6.00 | **<0.001** | 6.28 | 6.21 – 6.34 | **<0.001** | 1.35 | 1.12 – 1.59 | **<0.001** |
| Stimulation Protocol (38.5-52°C) | -0.17 | -0.22 – -0.12 | **<0.001** | -0.14 | -0.19 – -0.09 | **<0.001** | 0.08 | -0.09 – 0.25 | 0.351 |
| Stimulation Protocol (42-52°C) | -0.26 | -0.31 – -0.21 | **<0.001** | -0.21 | -0.26 – -0.15 | **<0.001** | 0.34 | 0.17 – 0.51 | **<0.001** |
| Capsaicin (Yes) | -0.17 | -0.22 – -0.12 | **<0.001** | -0.05 | -0.10 – 0.00 | 0.082 | 0.34 | 0.17 – 0.51 | **<0.001** |
| Stimulation Order | -0.01 | -0.03 – 0.01 | 0.325 | -0.01 | -0.03 – 0.01 | 0.380 | -0.06 | -0.12 – 0.00 | 0.059 |
| Capsaicin (Yes)*Stimulation Protocol (38.5-52°C) | 0.10 | 0.03 – 0.17 | **0.006** | 0.06 | -0.01 – 0.13 | 0.083 | -0.06 | -0.30 – 0.18 | 0.623 |
| Capsaicin (Yes)*Stimulation Protocol (42-52°C) | 0.14 | 0.07 – 0.21 | **<0.001** | 0.05 | -0.02 – 0.12 | 0.182 | -0.07 | -0.31 – 0.17 | 0.574 |
| Observations | 72 | | | 72 | | | 72 | | |
| B, beta; CI, 95% confidence interval  ms, millisecond | | | | | | | | | |
