# Supplementary material for: Contact Heat Evoked Potentials Are Responsive to Peripheral Sensitization: Requisite Stimulation Parameters
Source: Front Hum Neurosci. 2020 Jan 10;13:459. doi: 10.3389/fnhum.2019.00459 (PMC6966714; doi:10.3389/fnhum.2019.00459)
Supplement: Supplementary file 3 [file Table_3.DOCX]

| **Supplementary Table 3:** Effect of stimulation protocol, capsaicin conditioning, and stimulation order on CHEPs amplitude parameters. | | | | | | | | | | | | |
| --- | --- | --- | --- | --- | --- | --- | --- | --- | --- | --- | --- | --- |
|  |  | **N2P2 Amplitude [uV]** | | |  | **N2 Amplitude [uV]** | | |  | **P2 Amplitude [uV]** | | |
|  |  | *B* | *CI* | *p-value* |  | *B* | *CI* | *p-value* |  | *B* | *CI* | *p-value* |
| (Intercept) |  | 3.50 | 3.30 – 3.71 | **<0.001** |  | 2.73 | 2.50 – 2.95 | **<0.001** |  | 2.87 | 2.61 – 3.13 | **<0.001** |
| Stimulation Protocol (38.5-52°C) |  | 0.19 | 0.04 – 0.34 | **0.015** |  | 0.38 | 0.20 – 0.57 | **<0.001** |  | -0.00 | -0.19 – 0.18 | **0.976** |
| Stimulation Protocol (42-52°C) |  | 0.53 | 0.38 – -0.68 | **<0.001** |  | 0.74 | 0.55 – 0.93 | **<0.001** |  | 0.30 | 0.11 – 0.49 | **0.003** |
| Capsaicin (Yes) |  | 0.07 | -0.08 – 0.22 | 0.365 |  | 0.10 | -0.09 – 0.29 | 0.296 |  | 0.02 | -0.16 – 0.21 | 0.823 |
| Stimulation Order |  | -0.12 | -0.18 – -0.07 | **<0.001** |  | -0.15 | -0.21 – -0.08 | **<0.001** |  | -0.10 | -0.17 – -0.04 | **0.003** |
| Capsaicin (Yes)*Stimulation Protocol (38.5-52°C) |  | -0.00 | -0.21 – 0.21 | 0.977 |  | -0.09 | -0.35 – 0.18 | 0.515 |  | -0.09 | -0.18 – 0.35 | 0.523 |
| Capsaicin (Yes)*Stimulation Protocol (42-52°C) |  | 0.07 | -0.28 – 0.14 | 0532 |  | -0.10 | -0.37 – 0.16 | 0.448 |  | -0.00 | -0.27 – 0.26 | 0.976 |
| Observations |  | 72 | | |  | 72 | | |  | 72 | | |
| B, beta; CI, 95% confidence interval  uV, microvolt | | | | | | | | | | | | |
