# Supplementary material for: Contact Heat Evoked Potentials Are Responsive to Peripheral Sensitization: Requisite Stimulation Parameters
Source: Front Hum Neurosci. 2020 Jan 10;13:459. doi: 10.3389/fnhum.2019.00459 (PMC6966714; doi:10.3389/fnhum.2019.00459)
Supplement: Supplementary file 4 [file Table_4.DOCX]

| **Supplementary Table 4:** Comparison of CHEPs N2 latency, calculated from 10 or 20 contact heat stimulations (N=6). | | | | | | |  |
| --- | --- | --- | --- | --- | --- | --- | --- |
|  | **N2 Latency [ms]**  **20 Stimuli** | | **N2 Latency [ms]**  **10 Stimuli** | | **Two Sample t-test** | | |
|  | *Mean* | *CI* | *Mean* | *CI* | *(t, df)* | *p-value* | |
| Stimulation Protocol (35-52°C) | 361 | 338 – 381 | 366 | 346 – 385 | (-0.37, 22) | 0.71 | |
| Stimulation Protocol (38.5-52°C) | 302 | 289 – 316 | 291 | 283 – 298 | (1.32, 22) | 0.20 | |
| Stimulation Protocol (42-52°C) | 268 | 261 – 276 | 265 | 255 – 276 | (0.36, 22) | 0.71 | |
| CI, 95% confidence interval; ms, millisecond; *t*, t-stastitic ; *df*, degrees of freedom | | | | | | |  |
