# Supplementary material for: Contact Heat Evoked Potentials Are Responsive to Peripheral Sensitization: Requisite Stimulation Parameters
Source: Front Hum Neurosci. 2020 Jan 10;13:459. doi: 10.3389/fnhum.2019.00459 (PMC6966714; doi:10.3389/fnhum.2019.00459)
Supplement: Supplementary file 5 [file Table_5.DOCX]

| **Supplementary Table 5:** Effect of stimulation protocol, capsaicin conditioning, and stimulation order on CHEPs N2 latency in additional six (6) participants. | | | |  |
| --- | --- | --- | --- | --- |
|  | **N2 Latency [ms]** | | | |
|  | *B* | *CI* | *p-value* | |
| *(Intercept)* | 5.94 | 5.84 – 6.03 | **<0.001** | |
| Stimulation Protocol (38.5-52°C) | -0.23 | -0.31 – -0.14 | **<0.001** | |
| Stimulation Protocol (42-52°C) | -0.37 | -0.45 – -0.29 | **<0.001** | |
| Capsaicin (Yes) | -0.15 | -0.23 – -0.07 | **<0.001** | |
| Stimulation Order | 0.01 | -0.02 – 0.04 | 0.576 | |
| Capsaicin (Yes)*Stimulation Protocol (38.5-52°C) | 0.11 | 0.00 – 0.23 | 0.059 | |
| Capsaicin (Yes)*Stimulation Protocol (42-52°C) | 0.16 | 0.05 – 0.27 | **0.010** | |
| Observations | 36 | | | |
| B, beta; CI, 95% confidence interval  ms, millisecond | | | |  |
